# Enzyme‐Loaded Nanoreactors Enable the Continuous Regeneration of Nicotinamide Adenine Dinucleotide in Artificial Metabolisms

**DOI:** 10.1002/anie.202012023

**Published:** 2021-02-25

**Authors:** Seong‐Min Jo, Frederik R. Wurm, Katharina Landfester

**Affiliations:** ^1^ Max Planck Institute for Polymer Research Ackermannweg 10 55128 Mainz Germany; ^2^ Sustainable Polymer Chemistry Group MESA+ Institute for Nanotechnology Universiteit Twente PO Box 217 7500 AE Enschede The Netherlands

**Keywords:** artificial metabolism, encapsulation, enzyme reactions, nanoreactors, nicotinamide adenine dinucleotide

## Abstract

Nicotinamide adenine dinucleotide (NAD) is an essential coenzyme for numerous biocatalytic pathways. While in nature, NAD^+^ is continuously regenerated from NADH by enzymes, all synthetic NAD^+^ regeneration strategies require a continuous supply of expensive reagents and generate byproducts, making these strategies unattractive. In contrast, we present an artificial enzyme combination that produces NAD^+^ from oxygen and water continuously; no additional organic substrates are required once a minimal amount pyruvate is supplied. Three enzymes, i.e., LDH, LOX, and CAT, are covalently encapsulated into a substrate‐permeable silica nanoreactor by a mild fluoride‐catalyzed sol–gel process. The enzymes retain their activity inside of the nanoreactors and are protected against proteolysis and heat. We successfully used NAD^+^ from the nanoreactors for the continuous production of NAD^+^ i) to sense glucose in artificial glucose metabolism, and ii) to reduce the non‐oxygen binding methemoglobin to oxygen‐binding hemoglobin. This latter conversion might be used for the treatment of *Methemoglobinemia*. We believe that this versatile tool will allow the design of artificial NAD^+^‐dependent metabolisms or NAD^+^‐mediated redox‐reactions.

## Graphical abstract

The right combination: An artificial enzyme combination is presented that continuously produces NAD^+^ from oxygen and water and a catalytic amount of pyruvate, without additional substrates and without generating byproducts. Three enzymes are covalently encapsulated into a substrate‐permeable silica nanoreactor by a mild fluoride‐catalyzed sol–gel process. NAD^+^ from the nanoreactors was successfully used for the continuous production of NAD^+^ for sensing glucose and for rapidly reducing the non‐oxygen binding methemoglobin to oxygen‐binding hemoglobin.

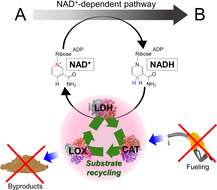

## Introduction

Nicotinamide adenine dinucleotide (NAD^+^; the oxidized form of NAD) is an attractive bio‐based oxidizing agent in synthesis, however, its regeneration from NADH (the reduced form of NAD) requires stoichiometric amounts of reagents or the use of organometallic catalysts. To date, no regeneration strategy that works without additional reagents in stoichiometric amounts has been reported. It is desirable to use NAD^+^ as an oxidizing agent only in small amounts and that the NAD^+^ can be regenerated throughout the synthesis. We demonstrate the continuous production of NAD^+^ by an artificial enzyme‐combination inside a substrate‐permeable and robust silica nanoreactor by water and oxygen as the necessary stoichiometric reagents with a minimal amount of pyruvate, without any additional substrates and without generating byproducts.

Living cells need a continuous supply of NAD^+^ but also have the capability to regenerate the NAD^+^.^[1]^ If the NAD^+^/NADH couple is used in synthetic chemistry for either oxidation or reduction, stoichiometric use of NAD^+^/NADH is necessary.^[2]^ A sustainable regeneration strategy of the coenzymes would also reduce the cost^[3]^ of natural or artificial metabolisms.^[4]^ Chemical, electrochemical, photocatalytic, and enzymatic strategies for both NAD^+^ and NADH regeneration have been proposed.[^3^, ^5^] Chemical regeneration of NADH by reducing agents such as NaBH_4_ is effective,^[6]^ but its high reactivity is problematic. Electrochemical regeneration is usually mediator‐dependent, side reactions to 1,6‐NADH or the NAD_2_ dimer have been described, and fouling of electrodes can occur.^[7]^ Photocatalytic regeneration became a recent focus that uses light as a green energy source but UV‐light might be harmful to some processes.^[8]^ Most methods for the NADH regeneration are non‐selective, and other redox‐sensitive compounds interfere.[^3^, ^5^]

In contrast, the enzymatic regeneration of NADH is the only selective method reported to date.[^3^, ^5^] However, the low stability of enzymes and their difficult isolation from a homogeneous reaction mixture are significant challenges. Encapsulation or immobilization of the enzymes is essential to increase stability and ease purification. However, it is accompanied by denaturation and reduced enzyme activity, for example, acidic/basic pH, non‐selective chemistry, or organic solvents.^[9]^

**Figure 1:**
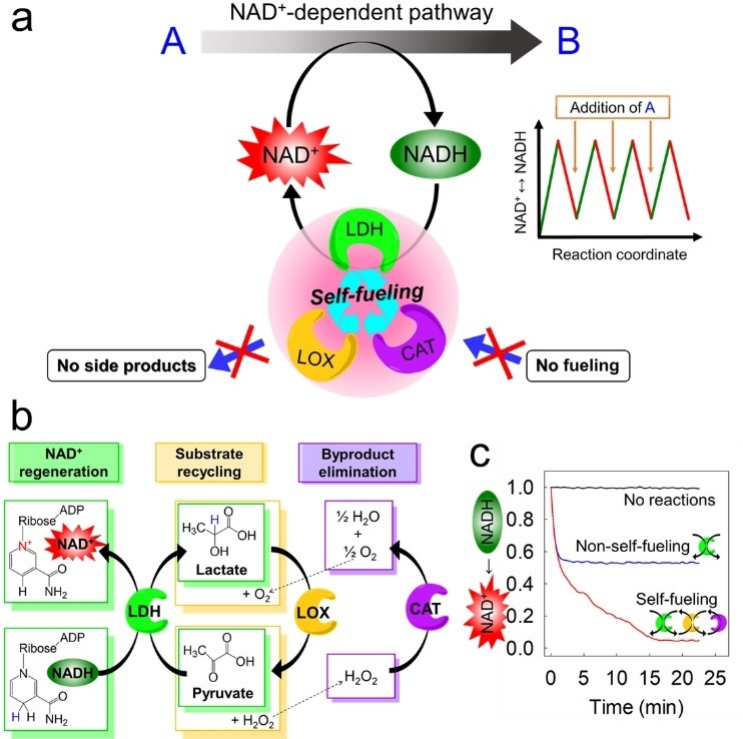
Continuous NAD^+^‐production and regeneration by an artificial enzyme set. a) The combination of LDH (lactate dehydrogenase), LOX (lactate oxidase), and CAT (catalase) encapsulated in semipermeable silica nanoreactors catalyze an NAD^+^‐dependent reaction A→B. b) Enzyme reactions inside the silica nanoreactor: LDH converts NADH to NAD^+^ and pyruvate to lactate. LOX recycles the pyruvate from lactate using water and oxygen and produces H_2_O_2_. CAT partially recycles H_2_O_2_ to water and oxygen. c) The reaction of lactate to pyruvate: if the pyruvate is added in nonstoichiometric amounts (NADH:pyruvate=2:1) the single enzyme (LDH) can oxidize 50 % of the NADH to NAD^+^ (blue curve), while the enzyme combination (LDH, LOX, CAT) achieves the full conversion (red curve).

We present a NAD^+^‐regeneration strategy that relies on three enzymes co‐encapsulated into semipermeable silica nanoreactors (SiNRs) prepared via a mild fluoride‐catalyzed sol–gel chemistry in the microemulsion. High encapsulation efficiencies with conserved enzyme activity were achieved. The artificial enzyme combination of lactate dehydrogenase (LDH), lactate oxidase (LOX), and catalase (CAT) only need a minimal amount of pyruvate to continuously produce NAD^+^ from NADH as the additional substrates oxygen and water are available in the mixture (Figure 1). The SiNRs were obtained as an aqueous dispersion and were combined exemplarily with the NAD^+^‐dependent i) fluorometric glucose detection and ii) the recovery of hemoglobin from methemoglobin. In combination with the possibility of reusing the SiNRs, the herein presented strategy for continuous NAD^+^ regeneration is an attractive strategy for the use in various NAD^+^‐dependent reactions.

## Results and Discussion

We realized the continuous production of NAD^+^ by the combination of lactate dehydrogenase (LDH), lactate oxidase (LOX), and catalase (CAT) encapsulated in a substrate‐permeable silica nanoreactor (Figure 1 a). From these enzymes, LDH produces NAD^+^ and lactate from NADH and pyruvate. The lactate is recycled by LOX to pyruvate without the need for a coenzyme or cofactor, producing hydrogen peroxide as the only byproduct (Figure 1 b), which is removed by disproportionation with CAT to oxygen and water. The enzyme cascade was initiated by the addition of NADH and a minimal amount of pyruvate to continuously produce NAD^+^ (if consumed by a reaction) (Figure 1 b). Reproduction of pyruvate by LDH/LOX, “self‐fueling”, enables the continuous NAD^+^ regeneration without the need to further supply pyruvate. The remaining substrates water and oxygen are present in the open aqueous system.

To investigate the compatibility of the artificial enzyme set, the NAD^+^ production was conducted in a solution containing LDH or a mixture of LDH, LOX, and CAT. When the ratio of NADH:pyruvate was set to 2:1, 50 % conversion of NADH to NAD^+^ by LDH alone was achieved, as pyruvate was converted to lactate. In contrast, the mixture of LDH, LOX, and CAT achieved the full conversion of NADH, which demonstrates the recycling of pyruvate by LOX (Figure 1 c).^[10]^

**Figure 2:**
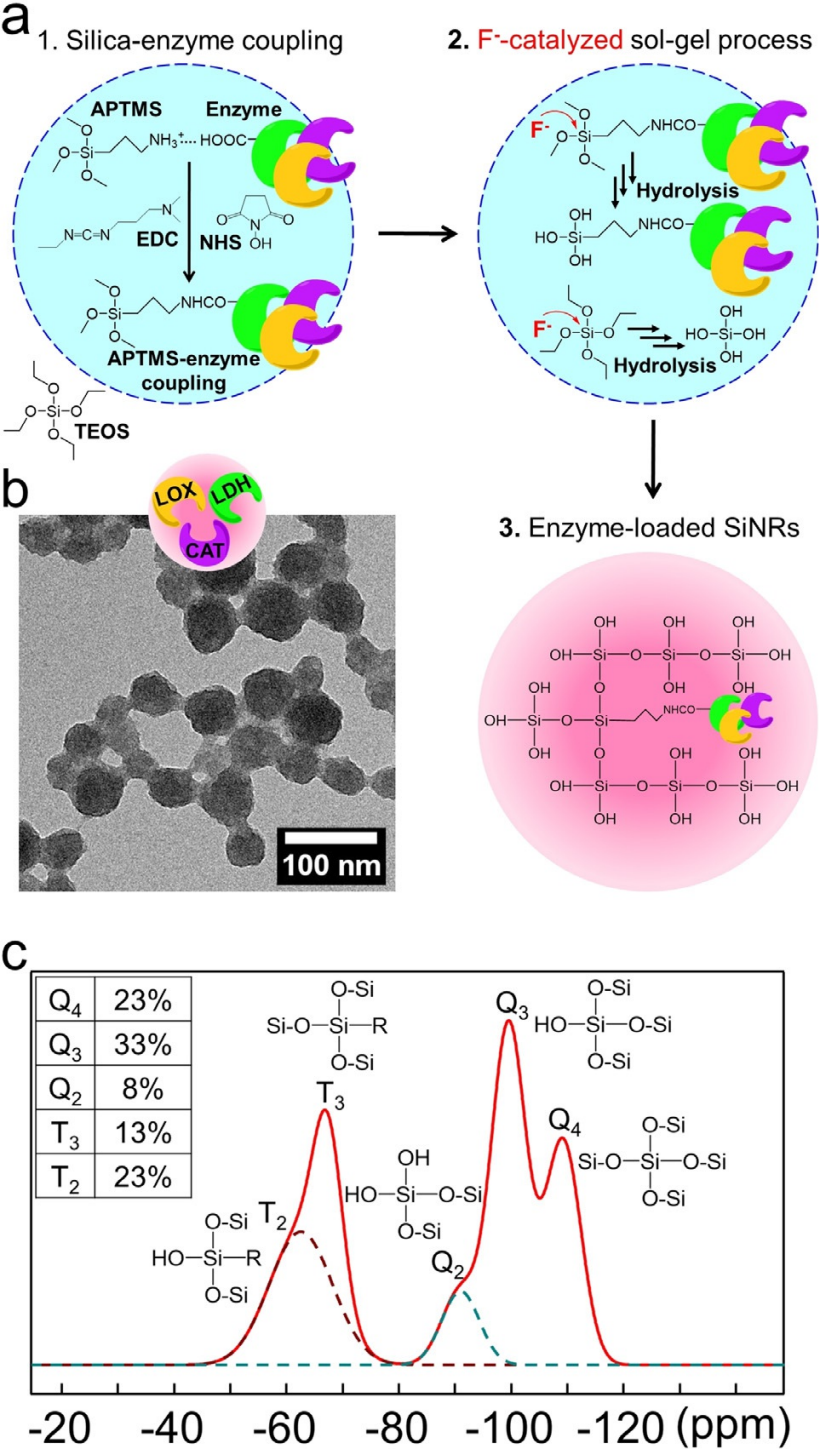
Preparation of silica nanoreactors (SiNRs) with covalently encapsulated enzymes. a) Fluoride‐catalyzed sol–gel chemistry and one‐pot procedure to enzyme‐loaded silica nanoreactors. b)  A transmission electron microscope image of enzyme‐loaded silica nanoreactors. c) Solid‐state ^29^Si NMR of LDH/LOX/CAT@SiNRs.

Subsequently, the three enzymes were covalently encapsulated into the substrate‐permeable silica nanoreactor by a mild sol–gel process (Figure 2 a). Unlike the conventional sol–gel process that relies on alkaline or acidic catalysts, we used fluoride (F^−^) as a catalyst to retain the enzyme activity (Figure 1 b).^[11]^ This artificial enzyme combination, confined in the same nanoreactor, enabled an efficient cascade reaction, modular handling, and easy separation from reactants with the possibility for reuse.^[12]^

Encapsulation of LDH, LOX, and CAT into the semipermeable silica nanoreactor was achieved by a fluoride‐catalyzed sol–gel process (Figure 2). As a control, a silica nanoreactor, loaded with LDH alone, was prepared. We co‐condensated tetraethyl orthosilicate (TEOS) as the matrix material with 3‐aminopropyl‐trimethoxysilane (APTMS) as a covalent anchor for the enzymes in a water‐in‐oil microemulsion with potassium fluoride (KF) as the catalyst, preserving the pH‐value at 7.4. During the process, the enzymes were covalently attached to the silica network by *N*‐(3‐dimethyl aminopropyl)‐*N′*‐ethyl carbodiimide hydrochloride (EDC) and *N*‐hydroxysuccinimide (NHS) chemistry. Circular dichroism (CD) spectra of enzymes in the fluoride solution show no significant change in their secondary structure (LDH and LOX for Figure S1), (CAT for the previous report^[13]^). Conventional sol–gel chemistry relies on acidic or alkaline catalysis, which may result in the denaturation of proteins and loss of enzymatic activity in some cases.^[11]^ For example, when ammonia was used as a catalyst for the condensation, no LDH enzyme activity was detected in the resulting nanoreactor (Figure S2). Besides, with the fluoride‐catalyzed sol–gel chemistry, a higher enzyme loading efficiency than conventional surface‐immobilization of enzymes was achieved (see below and ref. [14]).

As the LOX reaction produces hydrogen peroxide, a high amount of CAT is essential to disproportionate hydrogen peroxide into water and oxygen in the same nanoreactor and there is a need to protect LDH from the risk of oxidative denaturation. In the following, we adjusted the molar equivalents of the enzymes to LDH:LOX:CAT=3:1:15. The hydrodynamic diameters of the silica nanoreactors were determined by dynamic light scattering (DLS) to be between 200 and 270 nm (LDH@SiNRs: average diameter: 270 nm, PDI=0.404; LDH/LOX/CAT@SiNRs average diameter: 200 nm, PDI=0.420, Figures S3 and S4). From thermogravimetric analysis (TGA), a high enzyme loading was estimated by a weight loss of ca. 20 % organic contents in the self‐fueled nanoreactor during the thermal degradation compared to the empty nanoreactor (Figure S5). ^29^Si solid‐state NMR spectra showed the successful formation of the silica network by condensation of TEOS (Q_2_, Q_3,_ and Q_4_) and APTMS (T_2_ and T_3_) with a molar ratio of 2:1 by the sol–gel process (Figure 2 c). Besides, FT‐IR confirms the formation of silicon oxide by detecting two strong bands for Si‐OH (780 cm^−1^) and Si‐O‐Si (1040 cm^−1^) (Figure S6). Additionally, distinct amide vibrations at 1550 cm^−1^ were detected for the loading of enzymes. Measurements of the surface area of the nanoreactors by Brunauer–Emmett–Teller (BET) gas adsorption showed a BET surface area of 31.5 m^2^ g^−1^ and pore volume of 0.08 cm^3^ g^−1^, which was sufficient for the diffusion of small molecular substrates and products to the enzymes through the silica matrix.^[15]^

**Figure 3:**
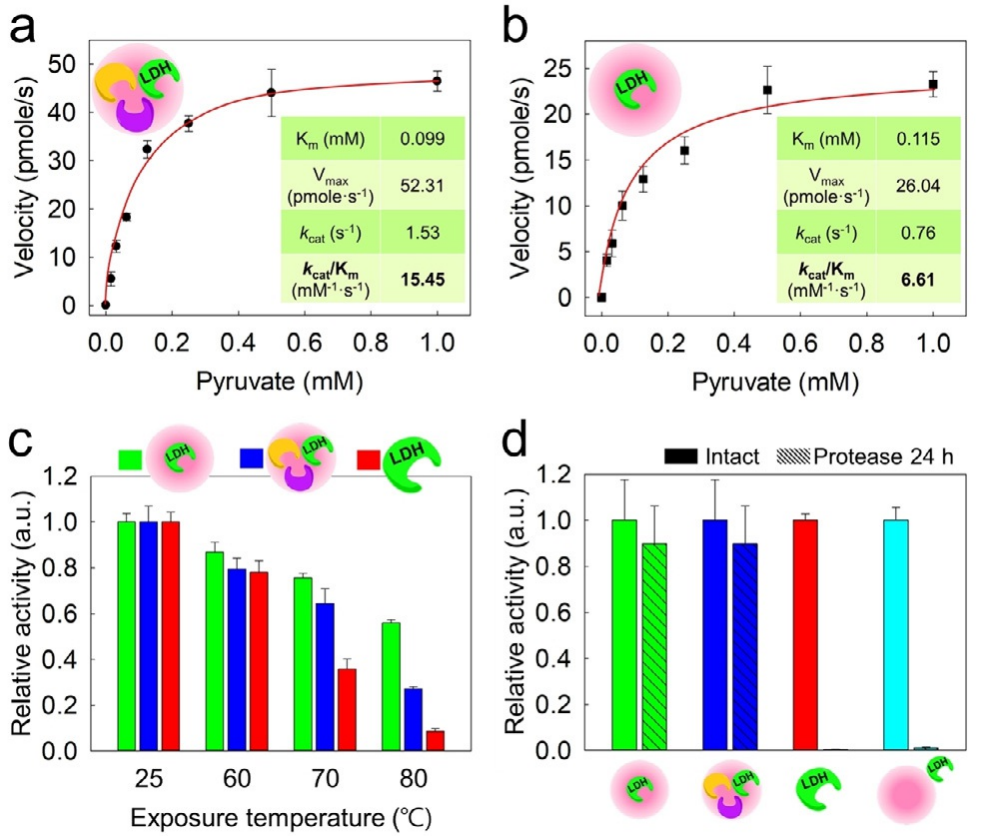
Enzyme reactions of silica nanoreactors. a,b) Michaelis–Menten kinetics of LDH/LOX/CAT@SiNRs (a), LDH@SiNRs (b) (LDH activity was measured). c) Thermal stability of encapsulated LDH and native LDH. d) Proteolytic stability of encapsulated, native, and surface‐immobilized LDH after incubation with Proteinase K.

All enzyme‐loaded silica nanoreactors exhibited a high enzyme activity, which is evident in the substrate‐permeability of the silica matrix. The enzymatic activity (*k*_cat_/*K*_m_) (with *k*_cat_=turnover number, *K*_m_=Michaelis–Menten constant) of LDH in the self‐fueled nanoreactors (LDH/LOX/CAT@SiNRs) was 2.3‐fold higher than that of the LDH@SiNRs for forward reactions (pyruvate + NADH → lactate + NAD^+^) (Figure 3 a,b). In particular, a two‐fold higher *k*_cat_ value was observed in LDH in the LDH/LOX/CAT@SiNRs. To explore the reason for the increased *k*_cat_ value, we investigated the product inhibition of LDH. According to the literature, the forward reaction of LDH can be inhibited by lactate as the product.^[16]^ Our results show a slightly decreased velocity for the forward reaction of native LDH (10 % for 1:20 of pyruvate:lactate), which is probably caused by the increasing amount of lactate in the mixture. Interestingly, much stronger effect of the product inhibition was observed for the LDH@SiNRs (25 % for a 2:1 of pyruvate:lactate ratio, Figure S7), presumably due to the local accumulation of the produced lactate inside the nanoreactors. In contrast, lactate was quickly eliminated by the reaction of LOX in the LDH/LOX/CAT@SiNRs, which we believe the main contribution to the increased *k*_cat_ value.

The activity of LOX did not differ remarkably for both single LOX‐loaded nanoreactors and LDH/LOX/CAT@SiNRs in terms of *K*_m_ and *k*_cat_ (Figures S8 and S9), because no substrate recycling (by LDH) occurred during the reaction.

We determined the changes of the enzymatic activity after exposure to 60 and 70 °C (below melting temperature (*T*_m_) of the native enzyme) and 80 °C (above *T*_m_) for 15 min (Figure 3 c). The enzymatic assay was performed at room temperature. The native LDH significantly lost its activity after heating to 80 °C (above *T*_m_) for 30 min to less than 10 % of the initial activity. In contrast, the encapsulated LDH showed much higher stability when heated to 80 °C for 2 h with ca. 40 % residual activity (Figure S10). Regarding such higher stability of the encapsulated LDH, previous studies have claimed that immobilized proteins by multipoint attachment could increase the resistance against heat, organic solvents, or denaturing agents, presumably due to the prevention of structural denaturation.^[17]^ It is consistent with our previous results that the encapsulated glucose oxidase and beta‐glucosidase in silica nanoreactors show higher stability than their native states.[^9^, ^14^] The encapsulated LOX also showed higher preserved activity than their native form at high temperatures. Unlike encapsulated LDH, the native LDH lost its activity at 70 °C (Figure S11). On the other hand, both native CAT and encapsulated CAT did not significantly lose their enzymatic activity after exposure to 80 °C for 15 min (Figure S12).

To further explore the structural stability of encapsulated enzymes, nano differential scanning fluorimetry (NanoDSF) measurements were performed.[^12^, ^18^] NanoDSF is based on changes in the intrinsic fluorescence of aromatic amino acids (i.e., Trp) in the protein structure during thermal denaturation. The folded and unfolded proteins exhibit a different emission ratio at 330 nm and 350 nm. We compared the structural stability of the enzymes in solution, surface‐immobilized, or encapsulated in silica nanoreactors. In the case of LDH, the initial emission ratios of 350 nm/330 nm were not significantly different between LDH in solution and the encapsulated LDH (0.82 and 0.83, respectively) (Figure S13), which indicated a similar folding state of native LDH in solution and the LDH after encapsulation. However, a significant difference between the pure LDH and the encapsulated LDH was detected after cooling to room temperature: while the native enzyme did not show any residual activity, the encapsulated enzyme in the silica matrix was still active, which might be attributed to partial refolding during the cooling procedure (Figure S14). According to the results, the encapsulated LDH recovered almost its initial fluorescence ratio of 350 nm/330 nm after the cooling, while native LDH did not.

For LOX, both the initial emission ratios at 350 nm/330 nm and melting temperature were similar between dissolved and encapsulated LOX (68 °C) (Figure S15). The stability of the encapsulated CAT was found to be more stable than native CAT (4 °C increased melting temperature in encapsulated CAT; Figures S16 and S17). This further underlines the mild conditions of the encapsulation procedure for a variety of enzymes.

Proteolytic resistance of encapsulated enzymes (LDH and LOX) in nanoreactors was investigated by exposure to Proteinase K (EC 3.4.21.64; 28.9 kDa): unlike the dissolved and surface‐immobilized enzymes, the nanoreactors with loaded enzymes did not lose their activity in the presence of Proteinase K (Figure 3 d for LDH and Figure S18 for LOX). This data further underlines the encapsulation of the enzymes and indicates that Proteinase K cannot penetrate the silica matrix due to its high molar mass. Additionally, no leakage of the enzymes from the nanoreactors was detected: the enzymatic activity remained unchanged after extensive washing of the dispersion, proving the covalent attachment of the enzyme inside of the silica matrix (Figure S19 and S20). In previous studies, only very little enzyme loading was achieved when the crosslinker (EDC, NHS) was omitted during the preparation of the silica nanoreactors.^[15]^

**Figure 4:**
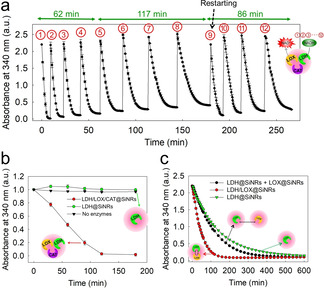
Continuous NAD^+^ production and regeneration. a) Continuous production of NAD^+^ by LDH/LOX/CAT@SiNRs upon the sequential addition of NADH. For each cycle, 3 mM NADH was used. After 8 additions, the SiNRs were centrifuged, washed, and reused. b) NAD^+^ regeneration with small amounts of pyruvate (2.5 μM), and NADH (0.25 mM). The normalized value of 1.0 along the y‐axis indicates the raw absorbance of 0.23. c) Comparison of the reaction kinetics of LDH/LOX@SiNRs, LDH/LOX separately‐encapsulated, and LDH@SiNRs. The starting concentration of NADH was 3 mM.

The continuous production of NAD^+^ was studied at different ratios of pyruvate: NADH at an initial molar ratio of pyruvate: NADH=2:1. The decreasing absorbance monitored the conversion of NADH into NAD^+^ at 340 nm. Additional NADH was added after the first equivalent was consumed. In the case of LDH@SiNRs, only two cycles produced NAD^+^; afterward, no oxidation of NADH was observed due to the lack of pyruvate and possible enzyme deactivation (Figure S21). In contrast, the self‐fueled nanoreactors (LDH/LOX/CAT@SiNRs) enabled the continuous regeneration of NAD^+^ for at least eight cycles (Figure 4 a). To initiate each cycle, only additional NADH was added to the dispersion, no further substrates or cofactors were necessary to produce NAD^+^. After eight cycles, the dispersion was centrifuged to separate the nanoreactors from the reactant. After re‐dispersion the nanoreactors in a new reaction cocktail, four additional NADH additions obtained the successful conversion to NAD^+^, which seems not to be the limit. Notably, the reaction velocity decreased from addition to addition (Figures 4 a and S22), which can be attributed to the changed NAD^+^ concentration in the reaction mixture that alters the equilibrium constant (*K*) after each addition of NADH and reduces the reaction rates (Figure S23). This was supported by conducting the NAD^+^ regeneration at different ratios of NADH:NAD^+^, proving a decreased conversion rate of NADH, when the amount of NAD^+^ was increased (Figure S24). After recycling the nanoreactors, the rate of the NAD^+^ production was increased to ca. 80 % of the initial velocity of the 1st cycle; probably only 80 % recovery was obtained due to loss of the material during the washing procedures (Figure S25). We confirmed the protection‐effect of CAT inside of the nanoreactors. The NADH oxidation was studied with a nanoreactor, loaded only with LDH and LOX (but without CAT). The reaction rates decreased gradually, and the NAD^+^ production ceased after the 6th cycle due to side‐reactions with hydrogen peroxide (Figure S26).

The efficiency of enzyme‐loaded nanoreactors was demonstrated by conducting the NADH oxidation at an extremely low pyruvate concentration, i.e., pyruvate:NADH=1:100, which would lead to only 1 % NAD^+^ conversion by the LDH, which resembles a change of 0.002 in the absorbance at 340 nm. Using the self‐fueled nanoreactors, almost quantitative conversion from NADH to NAD^+^ was achieved (Figure 4 b), proving that only a minimal amount of pyruvate was sufficient to produce NAD^+^, only relying on feeding with water and oxygen as substrates for the enzyme catalysis cascade (water and oxygen are present throughout the reaction cycles as the reaction proceeds under ambient air).

The confinement effect of several enzymes in the same nanoreactor was compared to separately encapsulated enzymes in different nanoreactors. We compared the kinetics for the NAD^+^ production for the LDH@SiNRs alone with two single enzyme nanoreactors (LDH and LOX) and the nanoreactors encapsulated LDH and LOX. Single LDH nanoreactors exhibited the lowest reaction rate to NAD^+^ as the concentration of pyruvate decreases during the process (Figure 4 c, green). The addition of LOX (in a separate nanoreactor) increased the reaction kinetics by a factor of ca. 1.1, as pyruvate was constantly regenerated by LOX (Figure 4 c, black). However, the confinement of both LDH and LOX in the same nanoreactors further increased the NAD^+^ regeneration by a factor of ca. 2.3 as the local pyruvate concentration remains high throughout the whole enzyme cascade (Figure 4 c, red).

**Figure 5:**
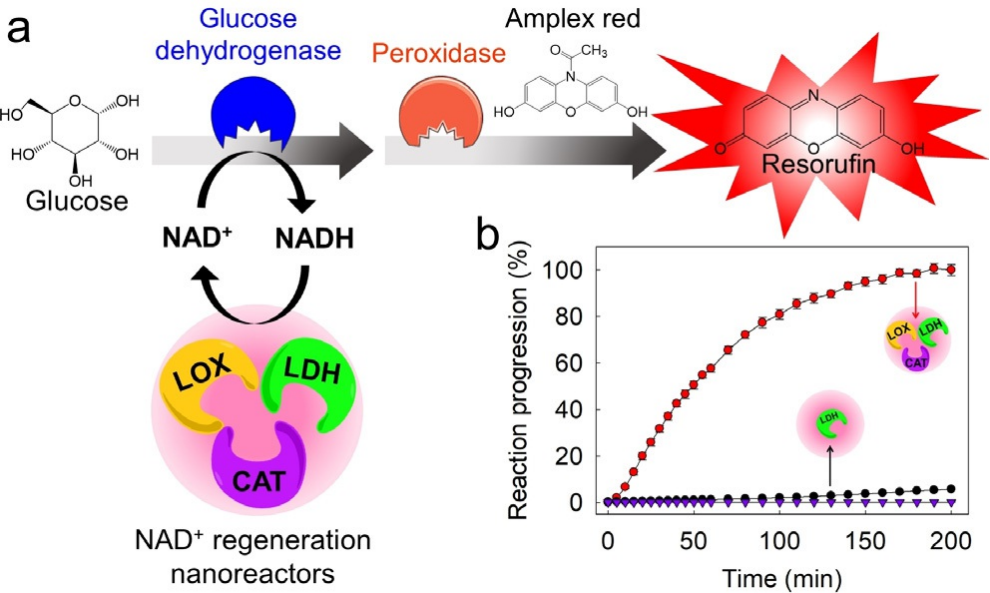
Utilization of LDH/LOX/CAT@SiNRs in NAD^+^‐dependent enzyme pathways. a) Glucose‐sensing: Glucose is a starting substrate for glucose dehydrogenase, producing glucono‐δ‐lactone. The peroxidase converts Amplex red into resorufin. Regeneration of NAD^+^ from NADH is necessary for the continuous progress of the reaction. b) Monitoring of the fluorescence of resorufin during artificial glucose metabolism using LDH@SiNRs or LDH/LOX/CAT@SiNRs. The molar ratio of NADH, pyruvate, and Amplex red was 1:1:10 (5 μM:5 μM:50 μM). The normalized value of 100 % at the y‐axis indicates the raw value for the fluorescence of 33 000.

The NAD^+^‐producing nanoreactors can be applied in artificial enzyme cascades, for example, for glucose detection.^[19]^ Glucose dehydrogenase is an NAD^+^‐dependent enzyme and catalyzes the conversion of glucose to glucono‐δ‐lactone. In a consecutive step, we used peroxidase, which converted Amplex red into resorufin, generating a fluorescence signal at 555 nm excitation and 595 nm emission, which is a simple glucose sensor (Figure 5 a). The molar ratio of NADH:pyruvate:Amplex red was 1:1:10, that is, a maximum of 10 % conversion would be achieved without NAD^+^‐regeneration. Using the NAD^+^‐producing nanoreactor, high conversion to resorufin was achieved (Figure 5 b). In contrast, when the LDH@SiNRs were used, only a low conversion (<10 %) was achieved, which was equivalent to the amount of NAD^+^ in the reaction mixture, as no continuous regeneration was possible. The results underline that the NAD^+^‐regeneration module can be combined with natural or artificial NAD^+^‐dependent enzymatic pathways, which opens the possibility for catalysis in artificial cells or reactors.^[20]^

**Figure 6:**
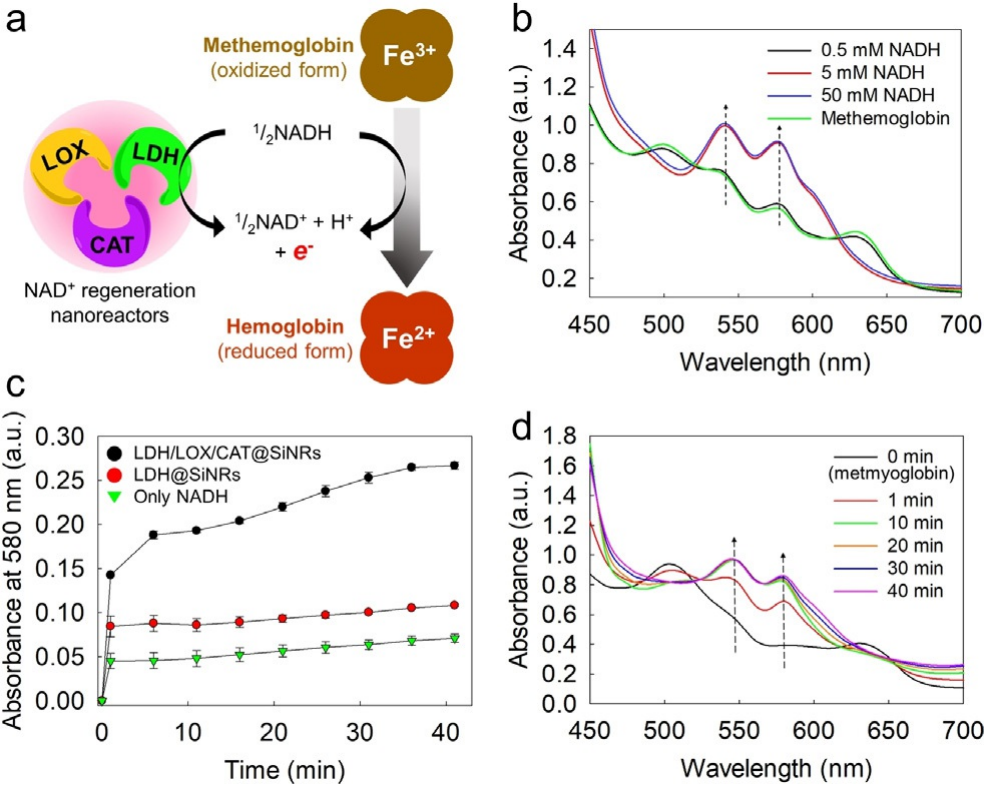
Rapid reduction of methemoglobin to hemoglobin with NAD^+^‐regeneration nanoreactors. a) A reaction scheme for the reduction of methemoglobin to hemoglobin. b) UV/Vis spectra showing the reduction of methemoglobin (8 mg mL^−1^; 125 μM) to oxyhemoglobin at different concentrations of NADH with LDH/LOX/CAT@SiNRs. c) Reduction of methemoglobin to oxyhemoglobin with 10 mM NADH by LDH/LOX/CAT@SiNRs, LDH@SiNRs, and only NADH. Absorbance at 580 nm indicates production of oxyhemoglobin. d) UV/Vis spectra showing the reduction of metmyoglobin to oxymyoglobin with 10 mM NADH by LDH/LOX/CAT@SiNRs.

With the redox potential of “NAD^+^+H^+^+2 e^−^→NADH” (−320 mV) and hemoglobin A (−52 to −71 mV),^[21]^ the NAD^+^‐regeneration module was also capable of reducing methemoglobin to hemoglobin. Hemoglobin is an essential protein in most vertebrates for oxygen‐transport, and it is a major component of red blood cells. The heme group is responsible for the binding and release of oxygen. *Methemoglobinemia* is a serious disease in which the hemoglobin is autoxidized to the methemoglobin, and thus it cannot bind to oxygen.^[22]^ In natural erythrocytes, the reduction of methemoglobin to hemoglobin is achieved by the NADH‐dependent methemoglobin reductase.^[23]^ The reduction of methemoglobin would be of high interest not only for natural red blood cells but also for the design of synthetic hemoglobin‐based oxygen carriers (Figure 6 a).^[24]^ As the oxidation of NADH to NAD^+^ by LDH is a two‐electron process, two heme groups might be involved to reduce Fe^3+^ into Fe^2+^. LDH/LOX/CAT@SiNRs were used to produce hemoglobin from methemoglobin (0.125 mM) by the help of NADH (5 and 50 mM). The UV/visible spectrum shows the formation of oxyhemoglobin (oxygen‐bound form): two strong absorbance bands at 540 nm and 580 nm, together with a decreased absorbance at 630 nm, which are typical for oxyhemoglobin, were detected (Figure 6 b).^[25]^ This finding suggested that our NAD^+^ regeneration system can be used for hemoglobin reconstitution, relying on NADH‐consumption and NAD^+^ production. Additionally, we demonstrated that the NAD^+^‐regeneration module (LDH/LOX/CAT) was able to produce hemoglobin from methemoglobin more rapidly at the early stage of the reaction, even though NADH alone would be able to produce hemoglobin gradually (Figure 6 c and Figure S27). The previous reports described the oxidation of NADH to NAD^+^ without catalysis triggers the reduction of Fe^3+^ to Fe^2+^,^[26]^ whereas much higher and faster methemoglobin reduction was obtained when the NAD^+^‐regeneration module was used in this study. We investigated the consumption of NADH for the reduction of methemoglobin: a rapid consumption of NADH was observed by the reaction of LDH/LOX/CAT@SiNRs (Figure S28). In contrast, when NADH was used alone or only LDH@SiNR was added, no significant change in the NADH concentration was detected. This implies that the reduction mainly relies on enzymatic reactions. These results add a new function to LDH/LOX/CAT@SiNRs and make it a promising replacement for methemoglobin reductase in artificial hemoglobin regeneration. It will open potential applications for treatment of *Methemoglobinemia*, combined with other oxygen carriers as hemoglobin reconstitution modules, and other enzyme‐mediated reactions.

In addition, the rapid conversion of metmyoglobin to oxymyoglobin was also possible using the LDH/LOX/CAT@SiNR in the presence of 10 mM NADH (Figure 6 d). A faster reduction of metmyoglobin by the use of the enzyme modules (1.76‐fold) was achieved when compared to hemoglobin. Myoglobin is a key oxygen carrier in the skeletal muscle tissue of many vertebrates and in almost all mammals.^[27]^ Unlike hemoglobin, myoglobin has a single heme group. Due to higher oxygen affinity than hemoglobin, myoglobin is an attractive oxygen carrier for blood substitutes.^[27b]^ Moreover, the prevention of myoglobin autooxidation is an important challenge in solving the problem of meat discoloration.^[28]^ Thus, these results could also possibly open a new avenue in the meat industry.

## Conclusion

An enzymatic NAD^+^‐regeneration nanoreactor was developed. The enzymes LDH, LOX, and CAT were encapsulated into the silica nanoreactors by fluoride‐catalyzed sol–gel chemistry retaining their enzymatic activities and stability. This “all‐in‐one” module allows their use in any NAD^+^‐dependent pathway, together with easy separation from the reaction mixture and the possibility of recycling. The nanoreactors, loaded with the three enzymes, operate without external fuelling or additional reagents through self‐recycling of all organic substrates. Only oxygen and water are needed. The NAD^+^‐regeneration was implemented into NAD^+^‐dependent pathways, e.g., artificial glucose metabolism. The NAD^+^‐regeneration module might be used in biocatalytic reactions or for the development of artificial mitochondria as artificial organelles in living or synthetic cells. Moreover, the self‐fuelled module can quickly recover the irreversibly oxidized methemoglobin (metmyoglobin) to the reduced hemoglobin (myoglobin), implying the potential for a new treatment to *Methemoglobinemia* and catalysing other redox reactions.

## Conflict of interest

The authors declare no conflict of interest.

## Supporting information

As a service to our authors and readers, this journal provides supporting information supplied by the authors. Such materials are peer reviewed and may be re‐organized for online delivery, but are not copy‐edited or typeset. Technical support issues arising from supporting information (other than missing files) should be addressed to the authors.

Supplementary
