# Supplementary material for: Enzyme‐Loaded Nanoreactors Enable the Continuous Regeneration of Nicotinamide Adenine Dinucleotide in Artificial Metabolisms
Source: Angew Chem Int Ed Engl. 2021 Feb 25;60(14):7728–34. doi: 10.1002/anie.202012023 (PMC8048563; doi:10.1002/anie.202012023)
Supplement: Supplementary file 1 — Supplementary [file ANIE-60-7728-s001.pdf]

Angewandte Chemie
Eine Zeitschrift der Gesellschaft Deutscher Chemiker

Supporting Information

Enzyme-Loaded Nanoreactors Enable the Continuous Regeneration of Nicotinamide Adenine Dinucleotide in Artificial Metabolisms

Seong-Min Jo, Frederik R. Wurm, and Katharina Landfester**

anie_202012023_sm_miscellaneous_information.pdf

Supplementary Information

Materials

List of reagents purchased from Sigma-Aldrich (Taufkirchen bei München, Germany): Tetraethyl orthosilicate (TEOS), lactate dehydrogenase recombinant from *E. coli* (LDH; EC 1.1.1.27, 96 unit/mg), catalase from bovine liver (CAT; EC 1.11.1.6, 3000 unit/mg) Triton X-100, Amplex red (Ampliflu™ Red), glucose (3:7 of α:β anomer), glucose dehydrogenase from *Pseudomonas sp.* (EC 1.1.1.47, 550 unit/mg), potassium fluoride, hemoglobin (methemoglobin form) from bovine, myoglobin from horse muscle, sodium lactate and hydrogen peroxide (35%).

List of reagents purchased from Carl Roth (Karlsruhe, Germany): EDC (1-ethyl-3-(3-dimethylaminopropyl) carbodiimide), sodium pyruvate, NADH (nicotinamide adenine dinucleotide, reduced form), NAD^+ (nicotinamide adenine dinucleotide), cyclohexane, *n*-hexanol, sodium phosphate dibasic and monobasic.

List of reagent purchased from Merck (Kenilworth, NJ, US): *N*-Hydroxysuccinimide (NHS) and APTMS ((3-aminopropyl)trimethoxysilane).

Other chemicals or enzymes: Lactate oxidase (LOX; EC 1.13.12.4, 101 unit/mg) was obtained from Sorachim (Lausanne, Switzerland). Horseradish peroxidase (EC 1.11.1.7, 300 unit/mg) was purchased from Thermo Scientific (Waltham, Massachusetts, US). Proteinase K (PK; EC 3.4.21.64, 2 unit/mg) was purchased from Calbiochem (San Diego, CA, US).

Methods and syntheses

Preparation of self-fueled modules

Preparation of enzyme-loaded silica nanoreactors. A water-in-oil (w/o) microemulsion was prepared as follows: cyclohexane (2 mL), *n*-hexanol (0.5 mL), Triton X-100 (0.45 mL) were mixed to prepare the organic phase. The aqueous phase (0.28 mL of a sodium phosphate buffer (10 mM, pH 7.4)) containing ATPMS (7.5 μL), NHS (2 mg), EDC (2 mg) and the respective enzyme(s) (LDH (1.2 mg) and/or LOX (0.4 mg) and/or CAT (0.1 mg)) were mixed and added to the organic phase. Under magnetic stirring (500 rpm), the coupling reaction was carried out for 2 h at room temperature. Then, TEOS (60 μL) and potassium fluoride solution (0.5 mM) were further added to the reactant, so that the final concentration of fluoride anion in the reactant was 0.1 mM. After 2 days of stirring (500 rpm, room temperature), the product was precipitated into ethanol (10 mL) and centrifuged (11000 rpm, 20 min), repeating twice. The pellet was dried at reduced pressure (yield = ca. 8.5 mg). The nanoreactor were redispered into buffer at a solid content of 16 mg/mL for further investigations. For the NH_3 catalyzed preparation of nanoreactor, 5% ammonia was used as a catalyst instead of potassium fluoride.

Preparation of surface-attached enzymes on nanoreactors. Bare nanoreactors were prepared as described above but without adding the enzymes, NHS, and EDC. For the conjugation of enzymes to the surface, bare nanoreactors (5 mg) were redispersed in a sodium phosphate buffer (pH 7.4, 10 mM) and reacted with enzymes (0.2 mg of enzymes (LDH, LOX, CAT, respectively)), EDC (0.5 mg) and NHS (0.5 mg)) for 8 h. After the reaction, the nanoreactors were purified by centrifugation, the supernatant was exchanged against fresh buffer, and enzyme activity were determined from nanoreactors and supernatant.

Enzyme assays

The absorbance or fluorescence during the enzyme assays was measured by a TECAN plate reader (Infinite 1000, Männedorf, Switzerland).

LDH: For the Michaelis-Menten kinetics, reaction cocktails were composed of phosphate buffer (pH 7.4, 50 mM), NADH (0.5 mM) and pyruvate (serially diluted from 2 mM). Solutions of native LDH (with 0.0153 units (i.e. 0.16 µg) or encapsulated LDH of 16 µg were used. The assay was started by adding pyruvate, and the changes in absorbance at 340 nm at intervals of 15 s were measured (a 96-well plate was used).

LOX: For the Michaelis-Menten kinetics, the reaction cocktails were composed of phosphate buffer (pH 7.4, 50 mM), Amplex red dye (3.3 μM), horseradish peroxidase (15 units/mL), and lactate (diluted from 20 mM). Native LOX of 0.2 units (2 µg) or encapsulated LOX of 10 µg were used. The assay was started by adding lactate, and measured the changes in fluorescence at excitation 555 nm/ emission 595 nm at interval of 15 s (96-well plate was used).

CAT: The method is described our previous report (https://doi.org/10.1021/acsami.0c05588), slightly modified for this study. Each stock solution was prepared as follows: hydrogen peroxide (100 mM), Amplex red solution in DMSO (2 mM), HRP (0.005 unit/mL), and CAT (native or encapsulated). The reaction cocktail was prepared by mixing hydrogen peroxide (10 μL) and amplex red (2 μL) in total 1 mL volume with sodium phosphate buffer (10 mM, pH 7.4). For CAT assay, HRP (2 μL) and CAT (2 μL) were added to the reaction cocktail (100 μL) and monitored changes in fluorescence (excitation 555 nm/emission 595 nm) at intervals of 30 s for 5 min by a TECAN plate reader. 96-well plate was used. In this assay, HRP oxidizes Amplex red to resorufin by consuming hydrogen peroxide. When CAT is present, conversion reaction of the Amplex red is competitive between CAT and HRP, thus the reaction velocity becomes slow.

Enzyme Leakage tests

The dried nanoreactors were dispersed into the phosphate buffer (pH 7.4, 10 mM) during 2 h (intact nanoreactors), centrifuged (1st supernatant), re-dispersed in the buffer for 2 h, centrifuged (2nd supernatant) and re-dispersed into the buffer (washed nanoreactors). The enzyme assay was carried out using the following condition in 96 well plates:

LDH: The reaction mixture was prepared by mixing NADH (4 μL of 60 mM stock solution), pyruvate (4 μL of 120 mM stock solution), and enzyme/ nanoreactor (1 μL) in 91 μL

buffer (50 mM, pH 7.4). Total volume of the assay was 100 μL. Pyruvate was added to initiate the reaction. The changes in absorbance at 340 nm were monitored at intervals of 30 s.

LOX: The reaction mixture was prepared by mixing lactate (10 μL of 150 mM stock solution), horseradish peroxidase (5 μL, 300 units/mL of stock solution) and Amplex red (0.33 μL of 1 mM stock solution in dimethyl sulfoxide) and enzyme/ nanoreactor (1 μL) in 85 μL buffer (50 mM, pH 7.4). The total volume of the assay was 100 μL. Lactate was added to initiate the reaction. The changes in fluorescence at excitation 555 nm/emission 595 nm were monitored at intervals of 30 s.

Thermal stability of enzymes

Each native enzyme (LDH: 0.2 mg/mL, LOX: 0.2 mg/mL, CAT: 0.2 mg/mL; 300 μL) or nanoreactor (0.8 mg/mL nanoreactors) was put into a 4 mL vial, and soaked into oil bath (60, 70 and 80 °C, respectively) for 15 min (for Figure 2c, S10, S11) or until 180 min (for Figure S9). The activity of each enzyme was determined by above mentioned protocols.

NanoDSF

Each enzyme sample (native enzymes 0.4 mg/mL in pH 7.4 and 10 mM phosphate buffer, nanoreactors: 20 mg/mL) was filled into a glass capillary (5 μL). The temperature was increased from 20 to 95 ℃ at 1 ℃/min. Changes in fluorescence at excitation (280 nm) and emission (330 and 350 nm) were monitored by a Prometheus nanoDSF instrument (Nanotempertech, München, Germany).

Proteolysis test

The native enzymes (LDH or LOX), surface-conjugated enzymes, and the nanoreactors with loaded enzymes were incubated with 1 mg/mL Proteinase K (2 units/mL) at 37 ℃ for 24 h under gentle shaking. The enzyme assay was carried out without removal of Proteinase K. (Note: as for the assay of the LOX activity, horseradish peroxidase is used (see enzyme leakage section above), we checked proteinase K sensitivity of the horseradish peroxidase. The horseradish peroxidase

was not losing the activity by incubation of proteinase K for 48 h. The enzyme assay of LDH and LOX were carried out following same protocol as described above (see enzyme leakage test section).

Other characterizations

For ^{29}Si solid-state NMR, FTIR, TGA, XRD, BET (Quantachrome, Florida, US), dried powder of nanoreactors were used. For nanoDSF (Prometheus, Nanotempertech, München, Germany), LDH (4 mg/mL) or LOX (2 mg/mL), CAT (0.5 mg/mL) of native enzymes solution, or nanoreactors (16 mg/mL of solid contents) was used. Glass capillary was filled with the solution or suspension, then, the temperature was increased from 25 to 90 °C with 1 °C/min. Changes in fluorescence at an excitation wavelength of 280 nm and emission wavelength of 350 nm and 330 nm were monitored. For CD analysis (JASCO, Easton, MD, US), 4 mg/mL of LDH solution in pH 7.4 phosphate buffer with 0.1 mM KF were used.

NADH regeneration test

NADH (3 mM), pyruvate (6 mM), each module (nanoreactor) (32 μg) were used for the regeneration study. First, the reaction mixture composed of NADH, nanoreactors and buffer was prepared by mixing 5 μg of NADH (60 mM), 2 μL of modules (16 mg/mL solid content) and 91 μL of phosphate buffer (pH 7.4, 50 mM), and the baseline was set at 340 nm absorbance. Then, the reaction was started by adding 2 μL pyruvate solution (300 mM of stock solution). The total reaction volume was 100 μL. The absorbance (340 nm) was monitored every 30 s. When the reaction seemed to reach a plateau value, further NADH (0.5 μL of 600 mM) was added to the same mixture and the absorbance was monitored again. After repeating eight times, the modules were purified by centrifugation (20000 rpm for 40 min), redispersed in the buffer, and performed regeneration experiment again with new reaction mixture same as before (NADH 3mM, pyruvate 6 mM). The concentration of NADH was determined by a calibration curve, from 0 mM to 3 mM; the curve is proportional against concentration.

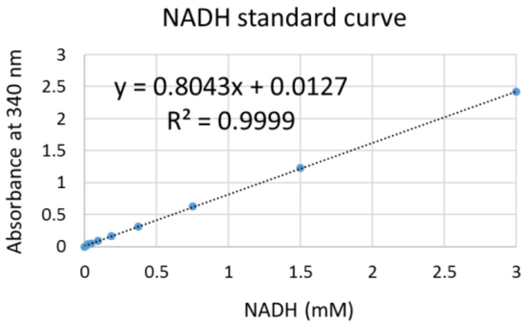

For the “low concentration” assay, 2.5 μM of pyruvate (1 μ of 0.25 mM stock solution), 0.25 mM of NADH (4.17 μL of 6 mM stock solution), and 2 μg of modules in the buffer (91.5 μL) were used. The changes in absorbance (340 nm) were monitored at intervals of 30 min over 180 min.

Glucose metabolism

The reaction mixture contained: pyruvate (5 μM), NADH (5 μM), glucose (2.4 mM), each module (32 μg), amplex red (50 μM), peroxidase (3 units), glucose dehydrogenase (5.5 units) in buffer (pH 7.4, 50 mM sodium phosphate) of 100 μL total volume. Amplex red (5 μL, 1 mM stock solution in DMSO), NADH (1 μL, 0.5 mM stock solution), glucose (2 μL, 120 mM), glucose dehydrogenase (2 μL, 0.5 mg/mL, 550 U/mg), horseradish peroxidase (2 μL, 0.5 mg/mL, 300 U/mg) and the modules (2 μL, 16 mg/mL) were added to sodium phosphate buffer (pH 7.4, 50 mM). To start the reaction, a pyruvate solution (1 μL, 0.5 mM stock) was added to the reaction mixture. The changes in absorbance at 340 nm was monitored every 60 s over a period of 200 min.

Reduction of hemoglobin/myoglobin

The mixture of hemoglobin (8 mg/mL), NADH (0 to 50 mM), pyruvate (1/10 molar amount against NADH for LDH/LOX/CAT@SiNRs, 1/1 molar amount against NADH for LDH@SiNRs,) and nanoreactors (80 μg) were used, 100 μL of total volume (50 mM, pH 7.4 buffer). NADH (5 μL, 1 M stock solution), pyruvate of (5 μL of 100 mM stock solution or 5 μL of 1M stock solution) and sodium phosphate buffer of (25 μL, 200 mM, pH 7.4) and deionized water (10 μL) were added to 50 μL of a methemoglobin solution (16 mg/mL). To start the reaction, 5 μL modules (16 mg/mL

stock) was added and then scanned the absorbance spectrum from 450 nm to 700 nm to get the reduction value. As control experiment, we checked changes in absorbance spectrum without LDH reaction that in absence of NADH or enzymes or pyruvate, respectively. There were no changes in absorbance in the presence of only pyruvate or only SiNRs. For myoglobin, we switched myoglobin instead of myoglobin.

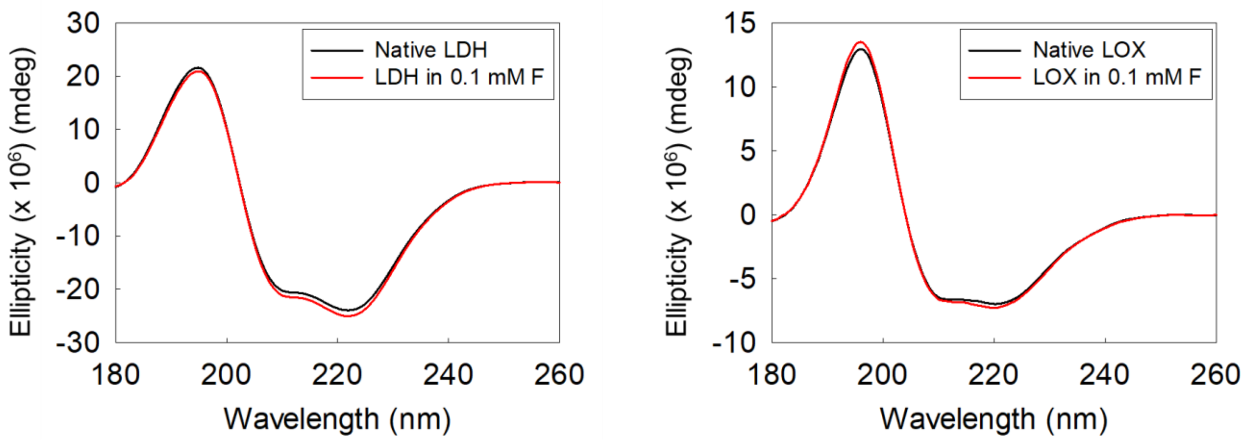

Figure S1. Circular dichroism (CD) spectrum of native enzymes (LDH, LOX) and enzymes (LDH, LOX) exposed to 0.1 mM potassium fluoride solution for 48 h at ambient temperature.

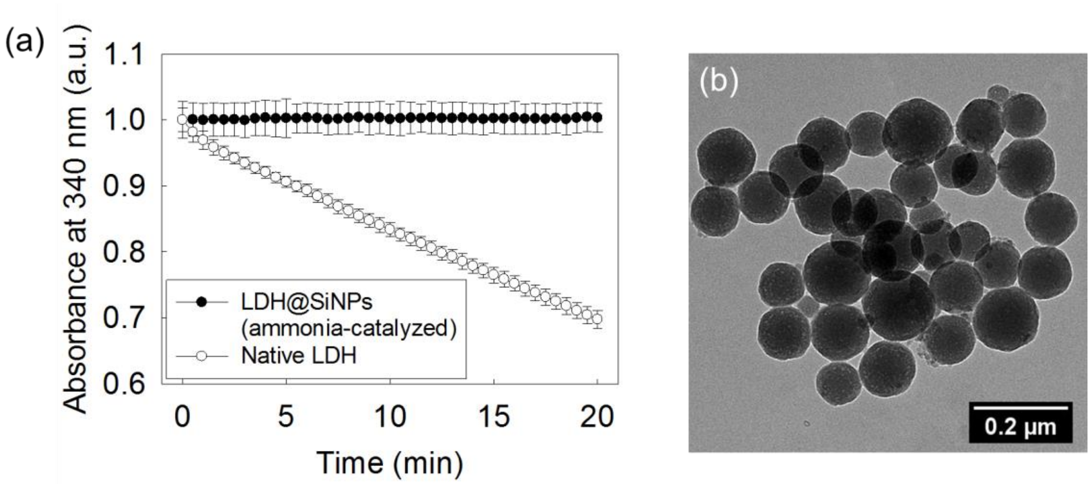

Figure S2. Enzymatic activity (a) and a TEM image (b) of LDH-loaded nanoreactor (SiNPs) that prepared by NH_3-catalyzed method.

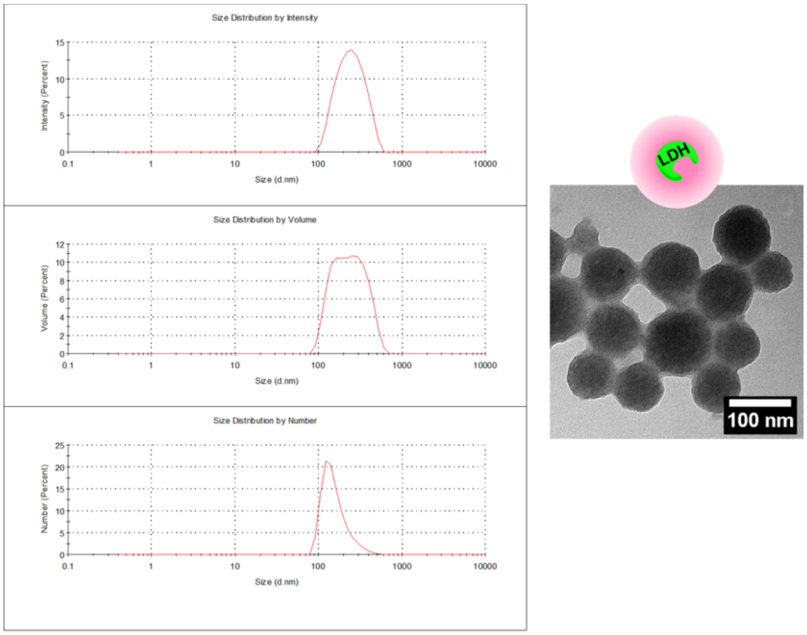

Figure S3. Size distribution (left) and TEM image (right; (magnification: x 50000)) of LDH-loaded nanoreactor (LDH@SiNRs).

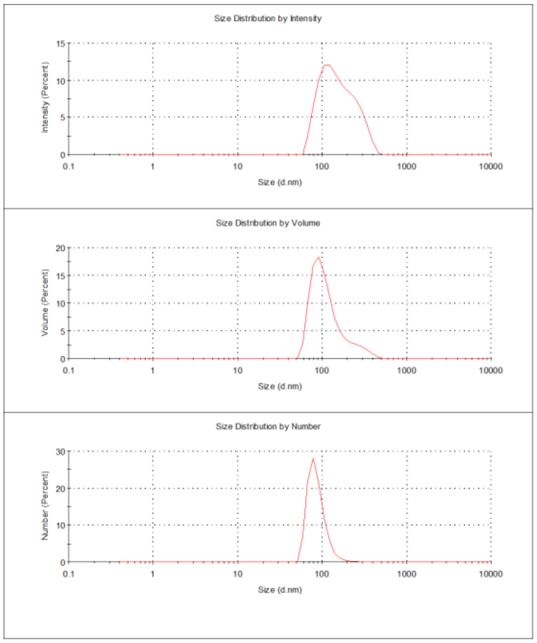

Figure S4. Size distribution of self-fueled nanoreactor (LDH/LOX/CAT@SiNRs).

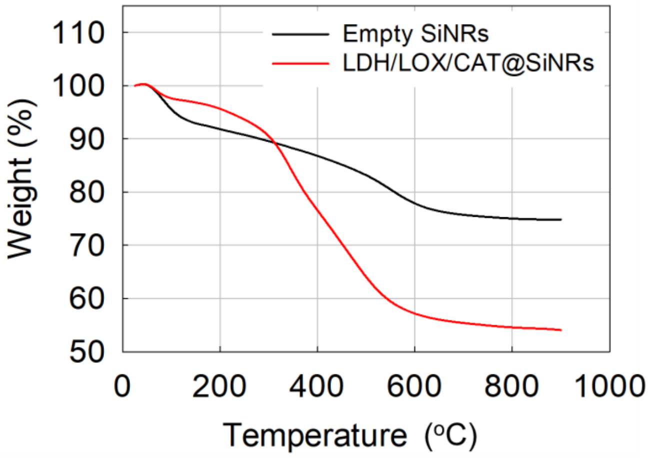

Figure S5. TGA for LDH/LOX/CAT@SiNRs and empty nanoreactor.

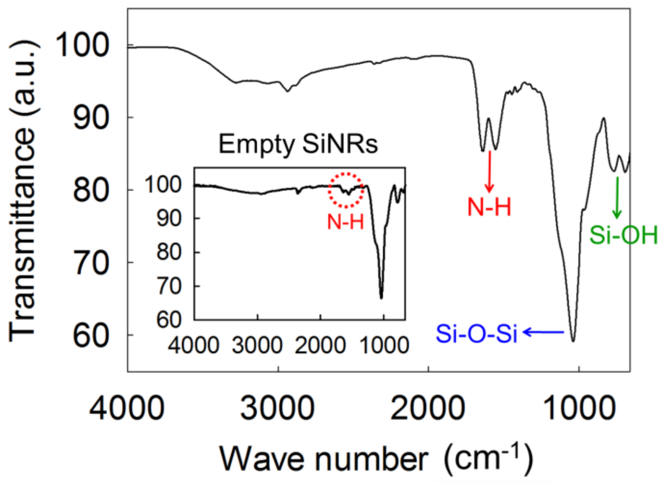

Figure S6. FTIR analysis of silica nanoreactor loaded with LDH, and bare nanoreactor (inset). Generation of little N-H bending on empty SiNR indicates in the presence of APTMS.

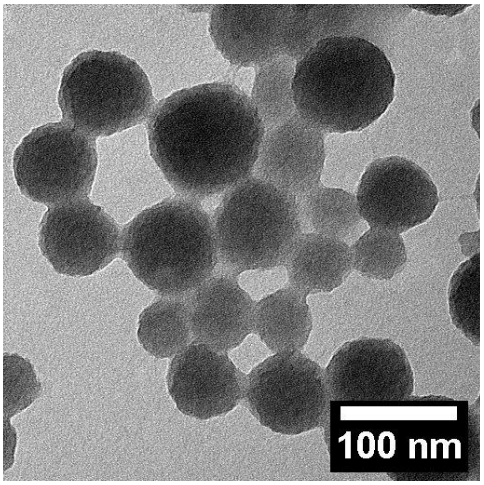

Figure S7. TEM micrograph of LOX-loaded nanoreactors (magnification: x 50000).

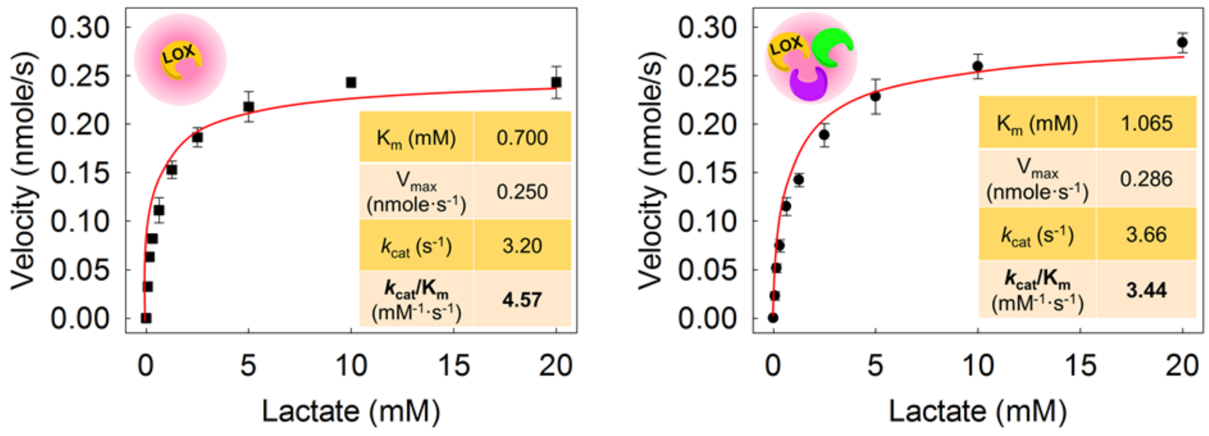

Figure S8. Michaelis-Menten kinetics of LOX@SiNRs (left panel) and LDH/LOX/CAT@SiNRs (right panel) in terms of LOX activity.

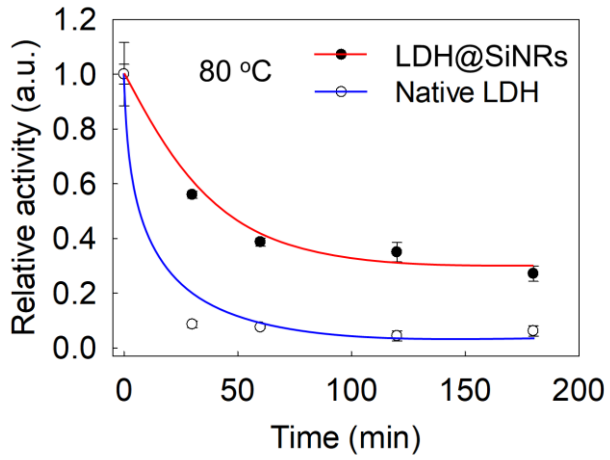

Figure S9. Enzymatic activity of native LDH and LDH-loaded nanoreactor after exposure to 80 °C over a period of 2 h.

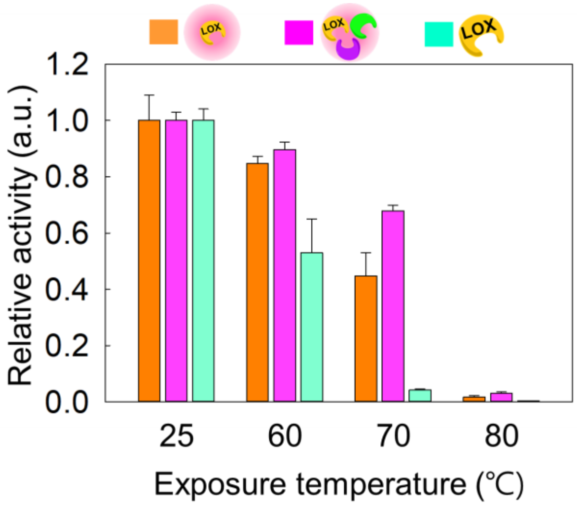

Figure S10. Enzyme activity of loaded LOX and native LOX after exposure of high temperature.

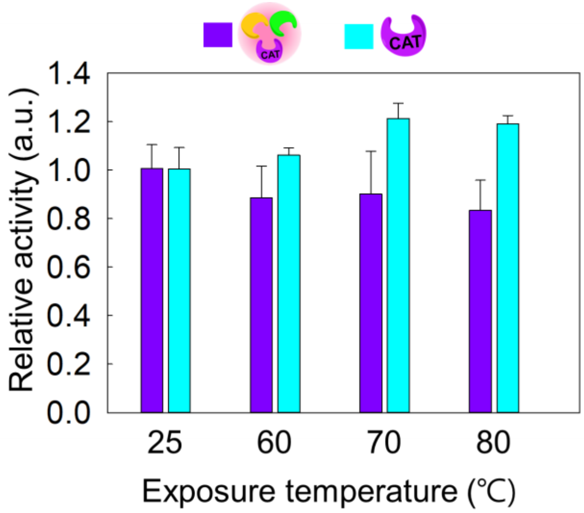

Figure S11. Enzyme activity of loaded CAT and native CAT after exposure of high temperature.

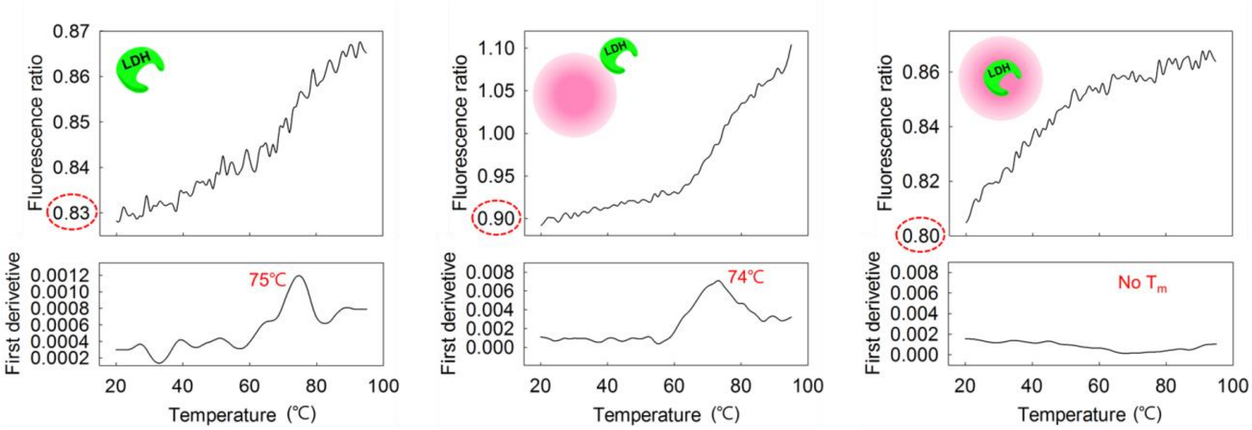

Figure S12. NanoDSF analysis of native LDH (left), surface-immobilized LDH on empty nanoreactor (center), and LDH loaded nanoreactor (right). Increasing intrinsic fluorescence indicates increasing fluorescent emission ratio of 350 nm/330 nm. Peaks on the Figures below indicate the melting temperature of the enzymes.

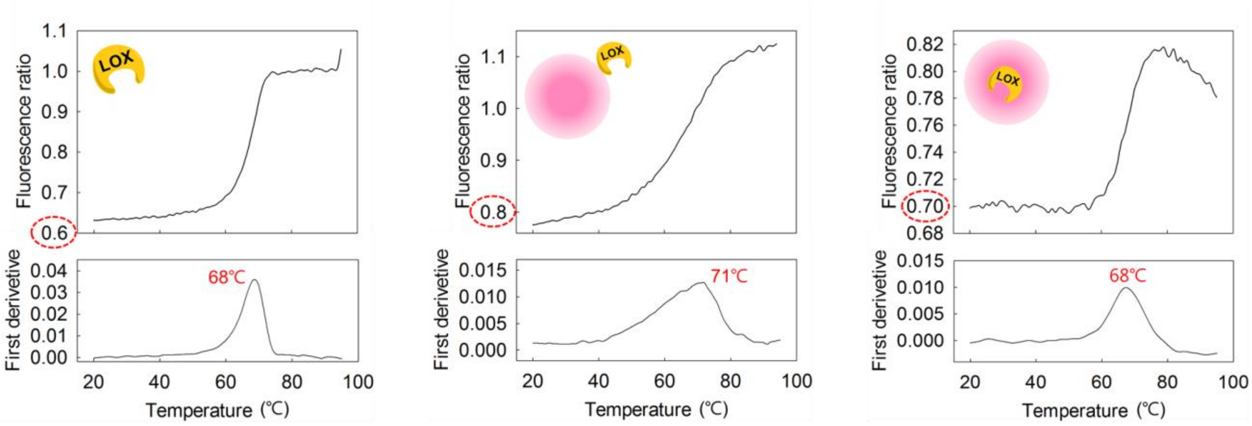

Figure S13. NanoDSF analysis of native LOX (left), surface-immobilized LOX on empty nanoreactors (center), and LOX loaded nanoreactors (right).

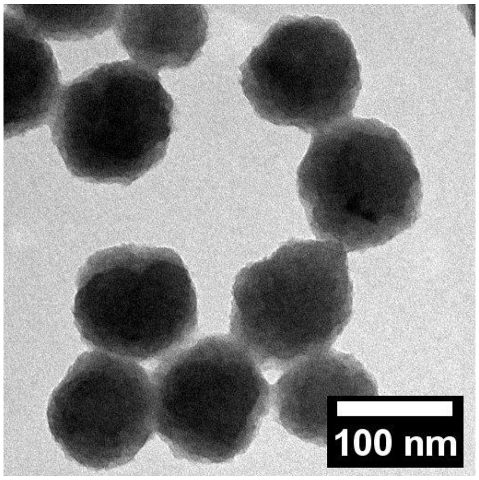

Figure S14. TEM micrograph of CAT-loaded nanoreactors (magnification: x 50000).

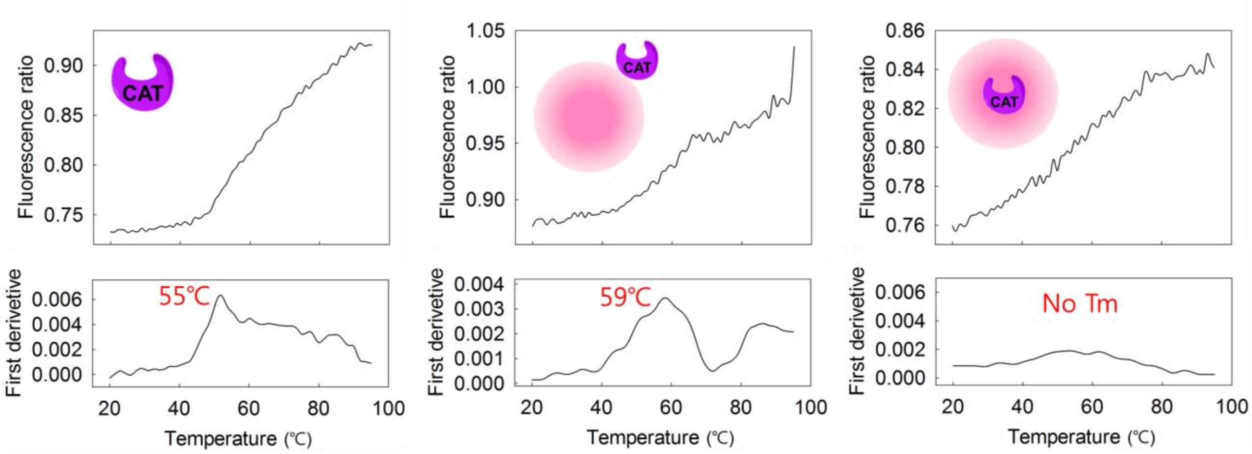

Figure S15. NanoDSF analysis of native CAT (left), surface-immobilized CAT on empty nanoreactors (center), and CAT loaded nanoreactors (right).

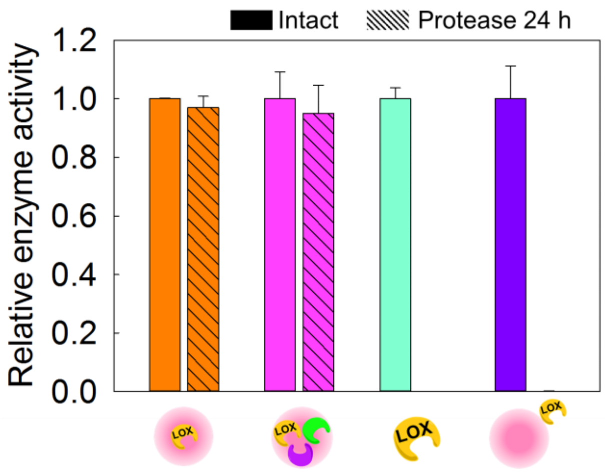

Figure S16. Protease stress resistance of loaded LOX in the nanoreactors, autopoietic modules, native LOX, and surface-immobilized LOX, respectively.

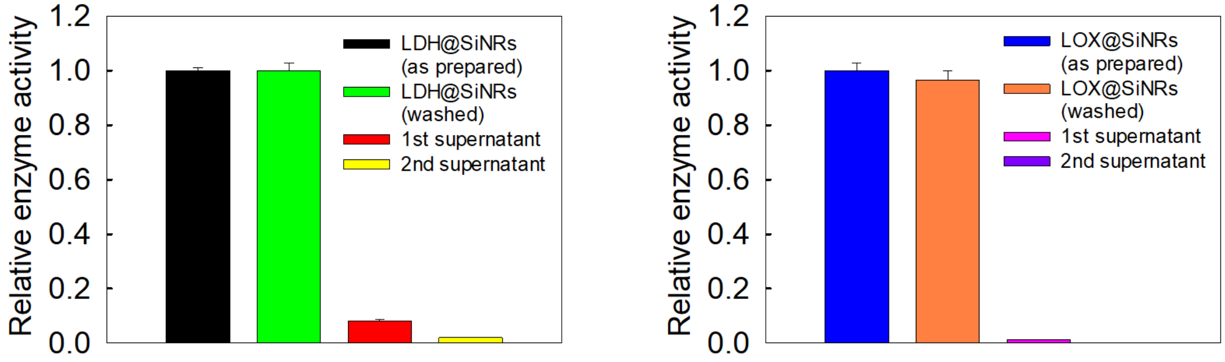

Figure S17. Enzyme leakage test of LDH@SiNRs (left) and LOX@SiNRs (right).

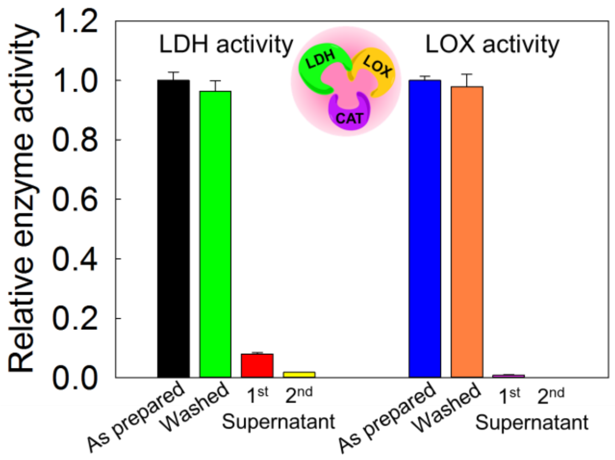

Figure S18. Enzyme leakage test of LDH/LOX/CAT@SiNRs in terms of LDH (left) and LOX (right).

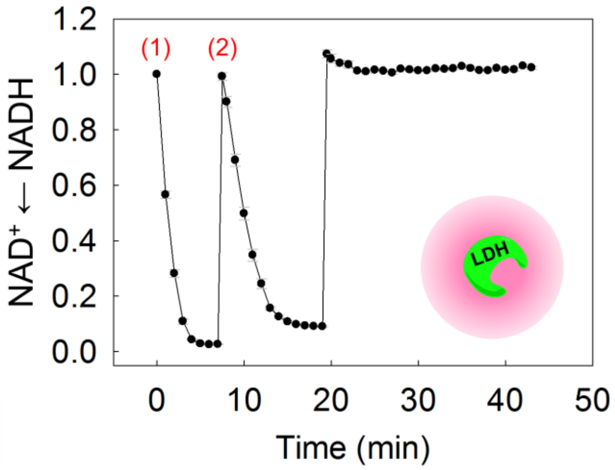

Figure S19. NAD^+ recycling test using non-self-fueled (LDH) SiNRs. The molar ratio of pyruvate and NADH is 2:1.

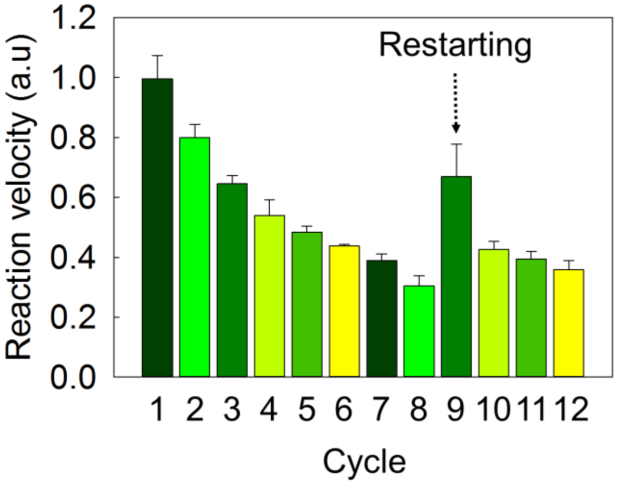

Figure S20. Initial reaction velocity in each cycle for the NAD^+ recycling by self-fueled (LDH/LOX/CAT) module (based on Figure 4A).

Equilibrium constant

Cycle	Eq. Constant (K)	Std dev
1	4347	570
2	1290	121
3	428	33.5
4	258	17.2
5	149	18.4
6	100	10.9
7	70	5.7
8	56	2.9
9 (restart)	440	58.2
10	181	15.8
11	113	7.5
12	78	2.0
Native LDH	4334	417

Figure S21. Equilibrium constants calculated in each cycle for NAD^+ recycling by self-fueled (LDH/LOX/CAT) module (based on Figure 4A).

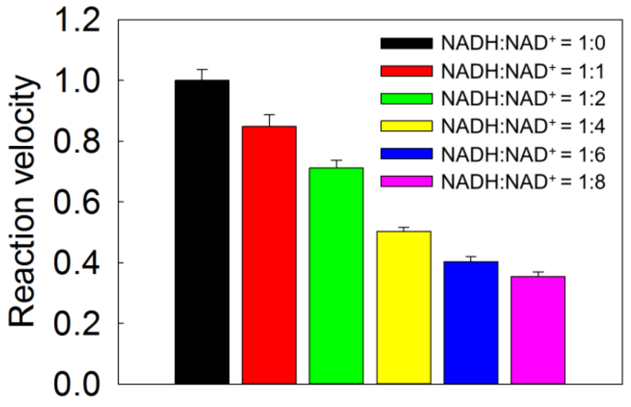

Figure S22. Effect of NAD^+/NADH ratio on LDH reaction.

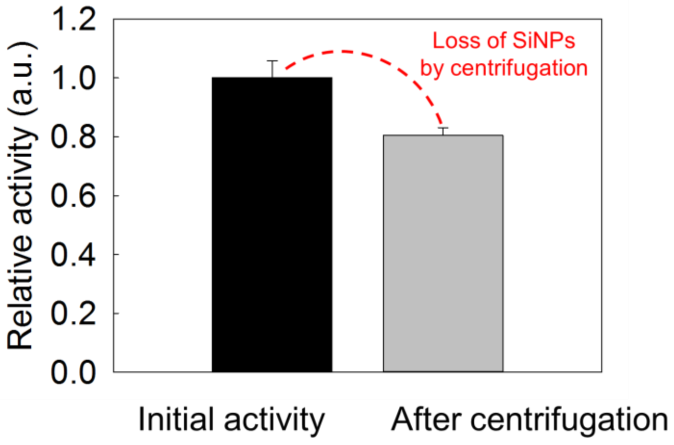

Figure S23. Loss of SiNPs by centrifugation. Compared is the enzymatic activity of LDH in LDH/LOX/CAT@SiNPs before and after centrifugation (20000 rpm, 40 min) indicating a loss of ca. 20% during the workup.

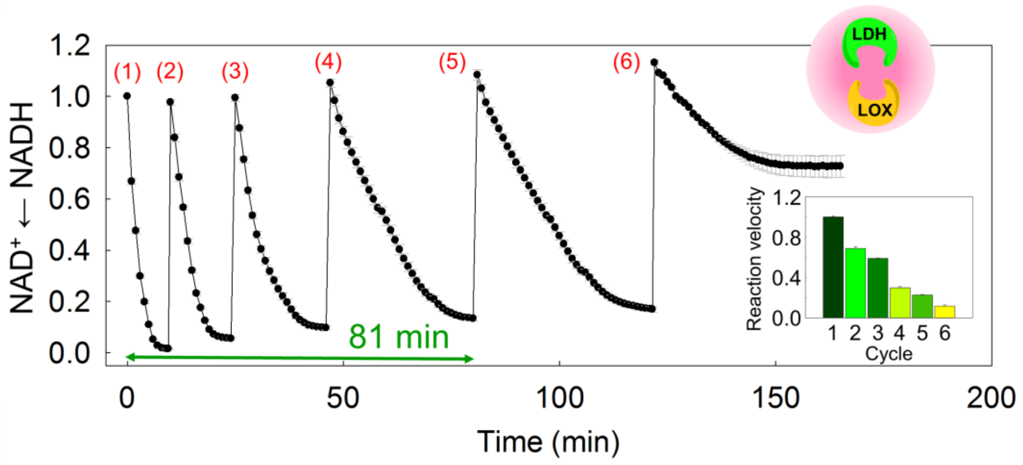

Figure S24. NAD^+ recycling test using LDH-LOX-loaded nanoreactors without CAT and reaction velocity (inset graph). The molar ratio of pyruvate and NADH is 2:1.
